# A self-adaptive method for creating high efficiency communication channels through random scattering media

**DOI:** 10.1038/srep05874

**Published:** 2014-07-29

**Authors:** Xiang Hao, Laure Martin-Rouault, Meng Cui

**Affiliations:** 1 HHMI Janelia Research Campus, 19700 Helix Drive, Ashburn, VA 20147, USA; 2 State Key Laboratory of Modern Optical Instrumentation, Zhejiang University, Hangzhou, 310027, China

## Abstract

Controlling the propagation of electromagnetic waves is important to a broad range of applications. Recent advances in controlling wave propagation in random scattering media have enabled optical focusing and imaging inside random scattering media. In this work, we propose and demonstrate a new method to deliver optical power more efficiently through scattering media. Drastically different from the random matrix characterization approach, our method can rapidly establish high efficiency communication channels using just a few measurements, regardless of the number of optical modes, and provides a practical and robust solution to boost the signal levels in optical or short wave communications. We experimentally demonstrated analog and digital signal transmission through highly scattering media with greatly improved performance. Besides scattering, our method can also reduce the loss of signal due to absorption. Experimentally, we observed that our method forced light to go around absorbers, leading to even higher signal improvement than in the case of purely scattering media. Interestingly, the resulting signal improvement is highly directional, which provides a new means against eavesdropping.

Controlling the propagation of electromagnetic waves has become the cornerstone of modern technologies^1^,^2^,^3^,^4^. Wave propagation in homogeneous or periodic structures has been extensively studied in the past^2^,^3^. In recent years, there have been intense research interest and efforts towards controlling wave propagation in random scattering media^5^,^6^,^7^,^8^,^9^,^10^,^11^,^12^,^13^,^14^,^15^,^16^,^17^,^18^,^19^,^20^,^21^,^22^,^23^,^24^,^25^,^26^,^27^,^28^,^29^,^30^,^31^,^32^,^33^. The apparent randomness can lead to unexpected physical consequences, such as the existence of open channels in strongly scattering media^19^. Utilizing time reversibility, scientists have explored focusing and imaging inside random scattering media for potential biomedical applications^7^,^8^,^9^,^11^,^12^. Many of the early ideas developed in the ultrasound research community^20^,^22^,^23^,^34^ have also been borrowed to solve the challenges in the photonics world. In this work, we propose a new scheme to control electromagnetic waves' propagation in random scattering media and explore its applications in optical wave communications.

Using optical wave as the information carrier in free space has become increasingly important in recent years^35^,^36^. The exceptionally large bandwidth promises to greatly improve the communication speed. Scattering and absorption are inevitable in optical wave propagation. We can treat the random scattering medium as a random matrix that performs transformation on optical waves^5^,^15^,^37^. Once the matrix is fully characterized, we can perform a singular value decomposition and figure out the channels with the highest transmission. However, measuring the scattering matrix is a daunting task in real world applications. A small area with 100 × 100 optical modes requires 10,000 wavefront measurements. The short correlation time of the scattering media (for example, air turbulence, cloud and fog) can easily invalidate the measured matrix.

In this work, we propose and demonstrate a robust and efficient method that takes only a few measurements to quickly establish high efficiency communication channels between two parties through a random scattering medium, regardless of the number of optical modes. We assume that the scattering matrix is completely random: each element of the matrix is a complex random number whose real and imaginary parts obey normal distribution. At such conditions, we can benefit from the results derived from random matrix theory. In particular, the statistical distribution of the eigenchannels' transmission values and the highest transmission value can be described by the Marchenko–Pastur distribution^38^,^39^. In Marchenko–Pastur distribution, there are a large percentage of channels of low transmission efficiency and a small percentage of channels of high transmission efficiency (as shown in Supplementary Fig. 1). We show in this work that if each party is equipped with a phase conjugation device, the wave can quickly converge to the highest transmission channels in a few iterations, as illustrated in Fig. 1a and the supplementary video. The idea is in fact rather straightforward. The transmission matrix is composed of a series of eigenchannels of different eigenvalues. Through an iterative phase conjugation, a monochromatic equivalent of the iterative time reversal utilized in the ultrasound applications^21^,^22^,^23^, the spectrum of the channels will be proportional to the initial eigenvalues at the power N, where N is the number of phase conjugation operations. Although we can tailor the channel spectrum in an exponential fasion, how fast the sytem converges depends on the eigenvalue distribution. In the extreme case where the distribution is bimodal and the eigenvalues are either 0 or 1, the system can converge in just one phase conjugation. In the most common cases where each matrix element is completely random, Marchenko–Pastur distribution applies, which is the focus of this work. In Supplementary Fig. 1, we used numerical simulations to illustrate the Marchenko–Pastur distribution and the convergence speed.

We employed the optical setup in Fig. 1b to experimentally test the rapid convergence onto the high efficiency channels. There are many ways to construct the phase conjugation devices. For example, we can combine a wavefront sensor and a MEMS deformable mirror or potentially fabricate a photonic phased array^4^ directly onto a CCD or CMOS sensor^40^. Here, we used a spatial light modulator (SLM) and a CMOS camera of identical pixel size to form a digital optical phase conjugation (DOPC) system^9^. We split the laser output with a 50/50 beam splitter and sent the two beams through two acousto-optic modulators (AOM) that provided frequency and power control of the diffracted light. We set the frequency difference between the two diffracted beams to 10 Hz such that we could record the optical wavefront by phase-shifting holography. The frequency difference (beating) can be seen as a continuous linear phase shifting over time. The camera ran at 40 fps and we converted the phase-shifting holograms to wavefront through a fast Fourier transform (FFT) in the time domain (*i.e.* determine the phase value of the 10 Hz beating for each pixel). At the start of the experiment, we opened the SLM shutter and closed the camera shutter of DOPC1 such that the light from DOPC1 illuminated the sample. At the same time, we closed the SLM shutter and opened the camera shutter of DOPC2 to record the optical wavefront. After the recording, we used the SLM of DOPC2 to generate the phase conjugation beam, which illuminated the sample for the second time. We iterated the process and observed the increase of the power transmission through the scattering medium. Four consecutive camera frames (0.1 second) were sufficient to yield the 2D wavefront. The entire iteration (five DOPC operations on each side) took 8 seconds that include the time used for recording the interferogram, calculating the wavefront by FFT, displaying the opposite wavefront on the SLM, turning on and off the shutters in the front of the SLM and the camera, and adjusting the laser beam intensity.

An ideal phase conjugation requires controlling both the amplitude and phase of the wave. In this work, we only controlled the phase of the wave and used a flat amplitude profile. Even without the amplitude control, we can still achieve 80% of the transmission enhancement with full control^31^, as shown in supplementary Fig. 2a. The transmission enhancement can be even higher if one party has less spatial modes than the other party^31^. However, the highest possible transmission is a monotonically increasing function of the spatial mode number and therefore it is of advantage to collect signal from more spatial modes (see supplementary Fig. 2b and 2c).

We performed both the transmission and the reflection experiments to test our method. Figure 2a is the simulated transmission enhancement. In only five iterations, the phase-only transmission enhancement reached ~2.7 that is ~70% of the highest possible power enhancement with both the amplitude and phase control^37^,^38^. Figure 2b–d are the experimental results of the transmission measurement and e–g are for the reflection measurement (setup shown in supplementary Fig. 3). In the transmission experiment, the scattering media were a stack of glass diffusers. In the reflection experiment, we sprayed TiO_2_ paint on a cover glass and the white color paint provided a diffusive reflective surface. We ensured that the scattering medium was sufficiently diffusive such that there was no ballistic component in the transmitted or reflected light. Both the convergence speed and the enhancement value agreed well with the simulation.

Will this method work for media with inhomogeneous absorbers and will it force the light to go around the absorbers? To answer these questions, we prepared absorptive objects and used them in both the transmission and the reflection measurements. The experimental results are summarized in Fig. 3. In the transmission measurement, we painted a cover slip with dark ink and inserted the cover slip between two glass diffusers. At the end of the five iterations, we removed one glass diffuser and the cover slip and directly imaged the light distribution. In the reflection measurement, we applied the dark ink onto the white color spray paint surface. At the end of the iterations, we replaced the sample with a white surface to examine the light distribution. In these experiments, we put the sample at ~0.7 m away from the DOPC such that the sample was near the Fourier space of the SLM, as shown in Supplementary Fig. 4. In both cases, we observed that light propagated around the absorbers and took the path with higher transmission or reflection. With the absorbers, the power enhancement can be even higher than in the purely scattering case, as shown in Fig. 3 and supplementary Fig. 5.

All optical measurements can benefit from a higher signal level. As pointed out in Shannon's pioneering work^41^, a communication channel's capacity is limited by the signal-to-noise ratio (SNR). For a shot-noise-limited system, a *N* fold signal improvement can increase the SNR by 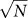. We performed the following two experiments to test how the increased transmission can improve the communication through random scattering media. The experimental setup is shown in supplementary Fig. 6a. We added a thermoelectrically cooled PMT detector (Hamamatsu H7422) with low dark noise (~20 count/sec) as the signal receiver. We placed a mask and a polarizer in front of the PMT such that it collected the same set of optical modes as the DOPC system. We used the AOM to modulate the light power to encode information. The signal dependence on the AOM driving voltage and the typical transmission enhancement data are summarized in Supplementary Fig. 6b–g. In the first experiment, we tested digital data transmission and encoded each pixel of an image as an 8-bit number and transmitted the binary data through the scattering medium (a stack of diffusers). Statistics showed that the enhanced transmission provided by our method decreased the bit error rate (BER), which indicates how often an error happens, from 4 × 10^−3^ to 1 × 10^−4^. We also tested analog data transmission and encoded the gray level of an image (Fig. 4a) as the optical power level. Zooming in on the details of Fig. 4b and 4c, we can clearly see the better image quality after the iterations.

An important feature of free space optical wave communication is the directionality. A third party who wishes to eavesdrop on the conversation has to find a way to access the light path between the two parties. By nature, scattering can spread the light beam in all directions, making eavesdropping possible. A key advantage of our method is that the enhancement is highly directional. Only the optical wave controlled by the two parties can expect signal enhancement. To demonstrate the anti-eavesdropping capability, we added another PMT to mimic the eavesdropping party (Supplementary Fig. 7a). Both the original PMT and the added PMT collected light from the same scattering surface. We chose proper optical attenuators to ensure that both PMT collected comparable amount of the diffusively reflected light prior to the iteration. We aimed to find out how the BER would change for both the receiving party and the eavesdropping party after the iterations, as shown in supplementary Fig. 7b. The experimental results (supplementary Fig. 7c and d) show that the eavesdropping party's BER remains constant while the receiving party can always enjoy a lower BER after the iterations. This feature can provide one additional protection against eavesdropping for more secure communications.

In summary, we report a self-adaptive method to create high efficiency communication channels between two parties through random scattering media. Different from the matrix measurement approach, our method did not attempt to measure the scattering media in any way. The statistic property of a completely random matrix described by the Marchenko–Pastur distribution can ensure a rapid convergence regardless of the number of optical modes, as shown in Supplementary Fig. 1. In our experiments, we controlled ~0.5 million optical modes at the best (see Supplementary Fig. 8). One would need 0.5 million wavefront measurements to determine the scattering matrix. In comparison, we only used five iterations (10 wavefront measurements) to quickly establish the high efficiency channels, several orders of magnitude faster than the matrix measurement approach. Besides random scattering, our method can force light to go around absorbers and achieve even higher signal enhancement. We tested our method with both digital and analog signal transmission tasks and demonstrated the much improved data quality through the high efficiency channels. An interesting feature is that the signal enhancement is highly directional. Only the communication between the two parties can be enhanced, which may potentially provide a new means to prevent eavesdropping. Although we mainly discuss the Marchenko–Pastur distribution, a commonly encountered case in real world applications, the same scheme can work for other types of distributions such as the bimodal distribution. The convergence can potentially be even faster, depending on the nature of the eigenvalue distribution. We expect our method to find important applications in optical or other short wavelength communications and fundamental research on random scattering systems.

## Supplementary Material

Supplementary Information SUPPLEMENTARY INFO

## Figures and Tables

**Figure 1:**
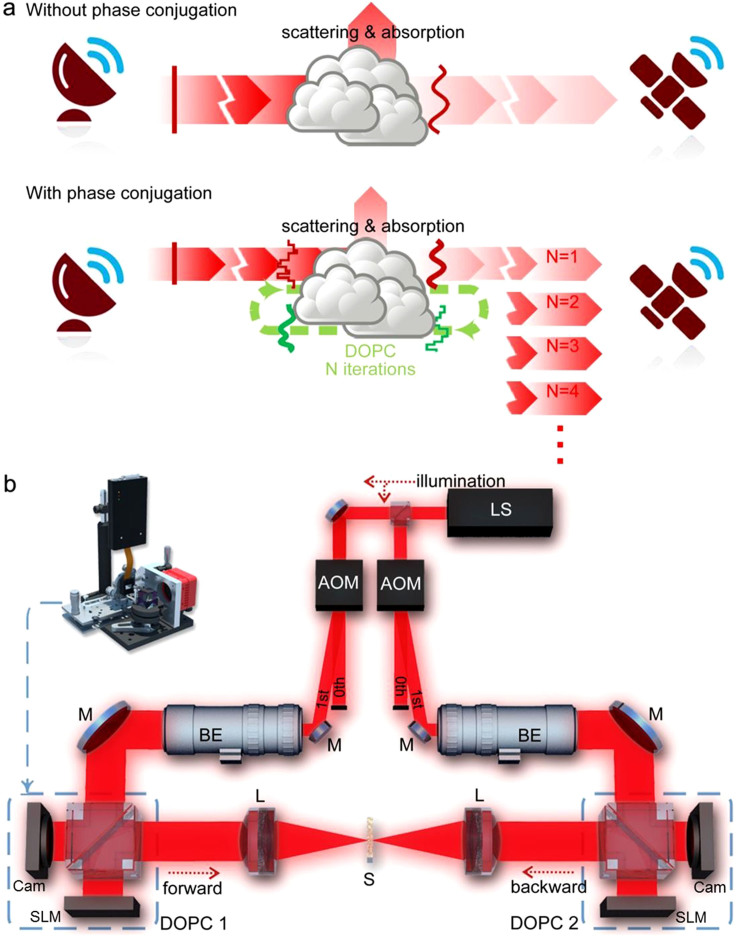
(a) Scheme of the self-adaptive method for creating high efficiency communication channels. Scattering and absorption can reduce the transmitted signals between the two parties. The iterative phase conjugation can force the light to converge onto the high efficiency paths and even go around absorbers. (b) Setup of the transmission enhancement measurement. LS, laser source (DL785-100-S, CrystaLaser, Nevada, USA); AOM, acousto-optic modulator (AOM-40AF, IntraAction, Illinois, USA); BE, beam expander that expands the beam diameter by 15; M, silver coated mirror; SLM, spatial light modulator (PLUTO Phase Only Spatial Light Modulator, Holoeye, Germany); Cam, camera (MV1-D2080-160-CL-12, PhotonFocus, Switzerland); L, lens (AC254-030-B, Thorlabs, New Jersey, USA). The sample was at the focus of the lens and the distance between the sample and the SLM (DOPC plane) was ~30 cm such that the light speckle was roughly two pixels in diameter on the SLM. All the setup drawings in this work were created by X.H. with Rhinoceros and Adobe Photoshop.

**Figure 2:**
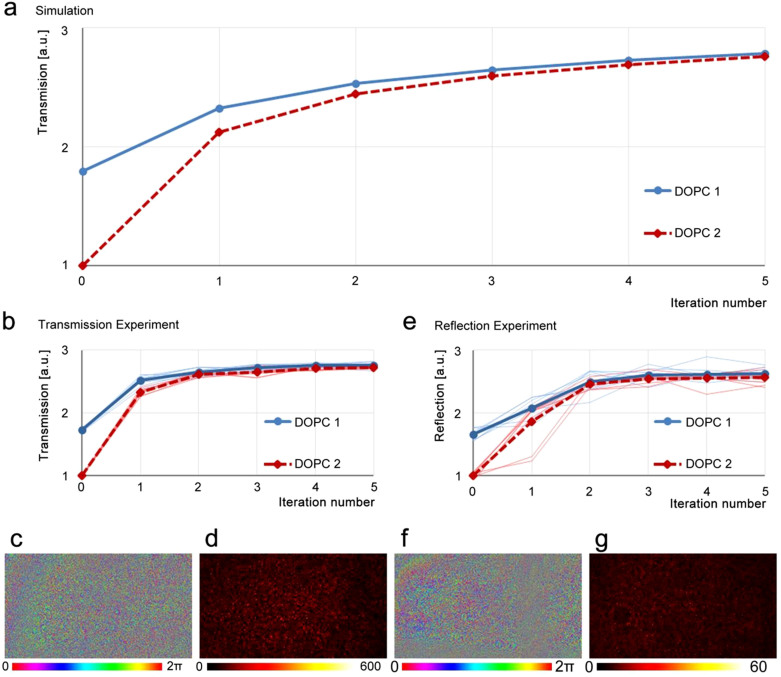
(a) Numerical simulation of the transmission and reflection enhancement in five iterations. (b–d) and (e–g) are the experimental results for the transmission and reflection configurations, respectively. (c) and (f) are the phase distributions on DOPC1 after five iterations, and (d) and (g) are the corresponding light intensity distribution on DOPC2.

**Figure 3:**
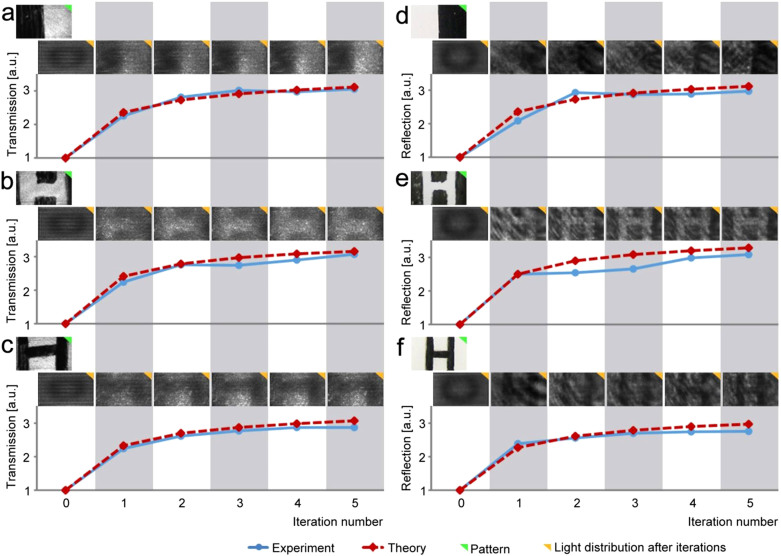
Absorber avoidance experiment. (a–c) are the transmission configuration data and (d–f) are the reflection configuration data. The light intensity distribution and the transmission/reflection enhancement after each iteration are shown in all the six data sets. The size of the sample was ~1 cm × 2 cm.

**Figure 4:**
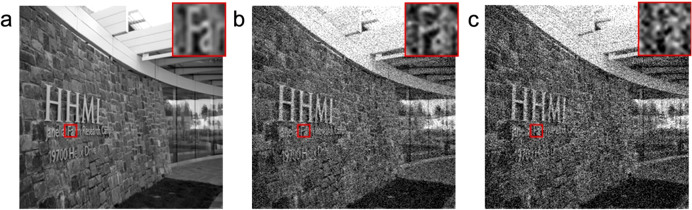
The analog image transmission experiment. (a) is the original image. (b) is the transmitted image after the iterations. (c) is the result before the iterations. The insets in image (b–c) provide a magnified view of the details for a clear comparison. We thank Dr. Tamir Gonen for providing the original image.
