# Supplementary material for: A self-adaptive method for creating high efficiency communication channels through random scattering media
Source: Sci Rep. 2014 Jul 29;4:5874. doi: 10.1038/srep05874 (PMC5376198; doi:10.1038/srep05874)
Supplement: Supplementary Information — SUPPLEMENTARY INFO [file srep05874-s1.pdf]

Supplementary information to

A self-adaptive method for creating high efficiency communication channels through random scattering media

Xiang Hao[1,2], Laure Martin-Rouault[1] and Meng Cui[1*]
[1]HHMI Janelia Research Campus, 19700 Helix Drive, Ashburn, VA 20147, USA
[2] State Key Laboratory of Modern Optical Instrumentation, Zhejiang University, Hangzhou, 310027, China

Supplementary video	Wavefront measured during the iteration and the corresponding transmission enhancement.
Supplementary Fig. 1	Marchenko–Pastur distribution and why rapid convergence is possible
Supplementary Fig. 2	Compare phase-only control and full control with numerical simulation and study the enhancement's dependence on the optical mode number
Supplementary Fig. 3	Experimental setup of the diffusive reflection measurement
Supplementary Fig. 4	Experiment setup of the absorber avoidance measurement
Supplementary Fig. 5	Supporting information of the absorber avoidance measurement
Supplementary Fig. 6	Supporting information of the image transmission measurement
Supplementary Fig. 7	Supporting information of the eavesdropping measurement
Supplementary Fig. 8	Speckle size measurement

Video: The wavefront variation during the iteration process and the corresponding transmission enhancement.

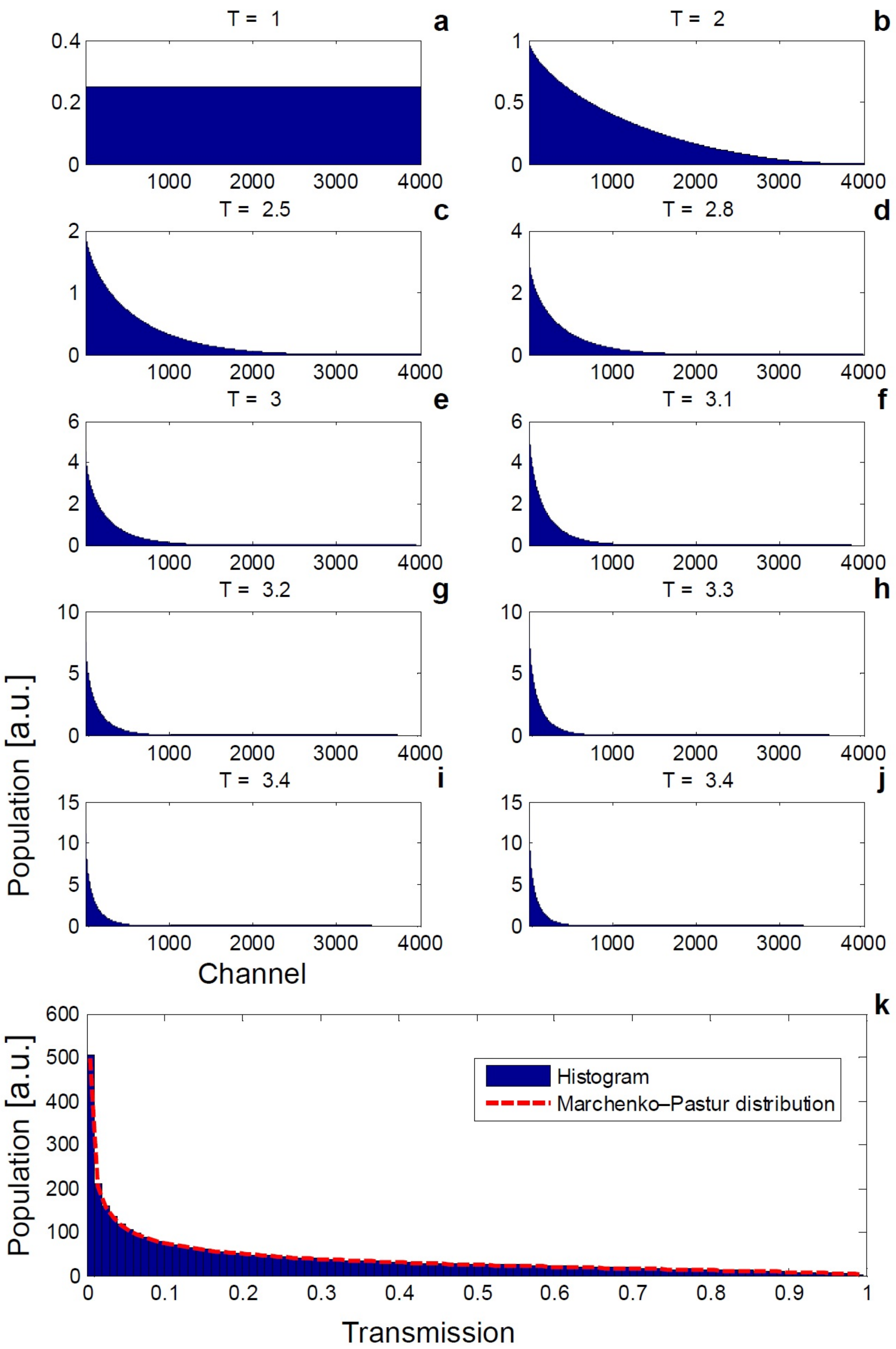
T = 1
a
T = 2
b
T = 2.5
c
T = 2.8
d
T = 3
e
T = 3.1
f
T = 3.2
g
T = 3.3
h
T = 3.4
i
T = 3.4
j
Population [a.u.]
1000 2000 3000 4000
Channel
k
Histogram
Marchenko–Pastur distribution
Population [a.u.]
0 0.1 0.2 0.3 0.4 0.5 0.6 0.7 0.8 0.9 1
Transmission

Fig. 1

The random scattering medium can be treated as a matrix that performs transformation on optical wave (a summation of optical modes), such that the input and output E field is described by $E_{output} = ME_{input}$. Each element of M is a complex number. For the open system studied in this work, there is no correlation between the elements of M. The real and imaginary parts of M follow normal distribution and thereby the Marchenko–Pastur distribution applies. We can perform a singular-value-decomposition on M such that we have $M = UtV^{\dagger}$ where t is a diagonal matrix composed of real and nonnegative values and t_i is the square root of the transmission value of the ith channel. The density of probability of t_i obeys the quarter-circle law ($\propto \sqrt{a^2 - t^2}(0 \le t \le a)$) when M is a square matrix, which is a special case of the Marchenko–Pastur distribution. The density of probability of the power transmission T ($T_i = |t_i|^2$) is therefore $\propto \sqrt{\frac{a^2 - T}{T}}(0 \le T \le a^2)$.

At the start of the iteration, the chances that the input light falls on any channel is uniform, as shown in **a**. After the first pass through the medium, the channels that have higher transmission will have higher population, as shown in **b**. In fact, **b** is identical to the power transmission spectrum of all the channels. The histogram of **b** (shown in **k**) is precisely described by the Marchenko–Pastur distribution ($\propto \sqrt{\frac{1-T}{T}}$, we normalize the highest transmission to 1). From **k**, we can see that a large number of channels have low transmission and a small number of channels have high transmission. As such, the medium behaves as a sharp high-transmission-pass filter that makes a rapid convergence possible. Through more iterations (**c-j**), we can see that the population quickly converges to the channels of high transmission and the overall transmission increases from 1 to 3.4 (85% of the maximum value 4) in five iterations. A 4000×4000 elements random matrix is used in all the above plots.

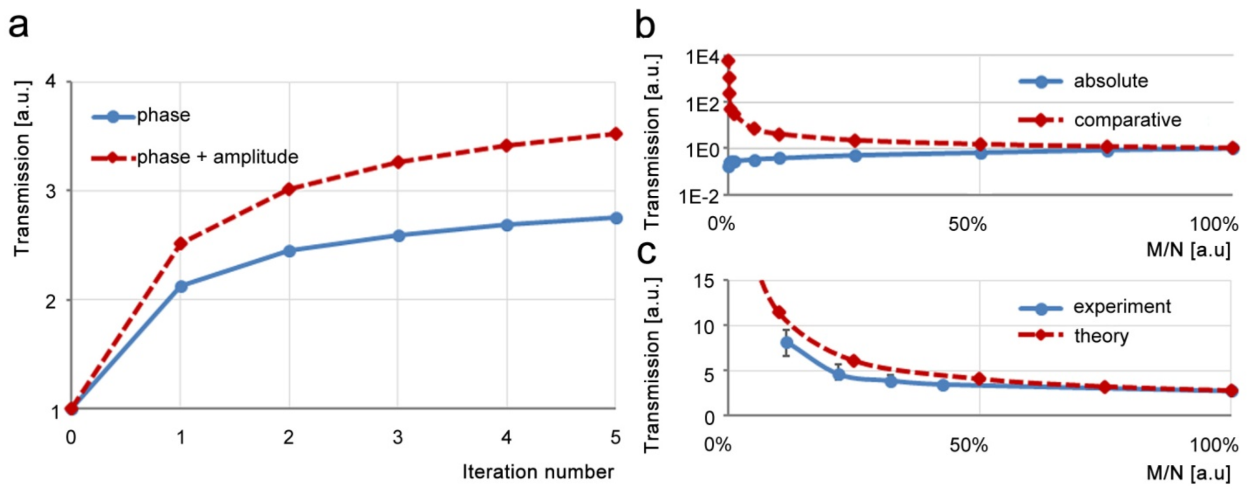

Fig. 2

We used numerical simulations to compare the transmission enhancement made by the phase-only control and the full control (phase + amplitude) for five iterations. The transmission enhancement is ~20% higher with the full control[31], as shown in **a**. Reducing the number of spatial modes on the output side of the medium can make the enhancement even higher. An extreme case is that there are N modes on one side of the medium and only one mode on the other side. The enhancement of the transmission to the one-mode side can be N fold[31] while the enhancement of the transmission to the N mode side is one (no improvement). For real world applications, we care about the absolute power transmission. Our simulation shows that it is advantageous to control as many modes as possible because the total collected power is a monotonically increasing function of the mode number. The enhancement ratio (red) and the total power transmission (blue) curves are summarized in **b**. We performed experiments to test the relation between the enhancement and the mode number by using an adjustable slit to block the area on the SLM. **c** shows the experimental result, which is reasonably close to the simulation.

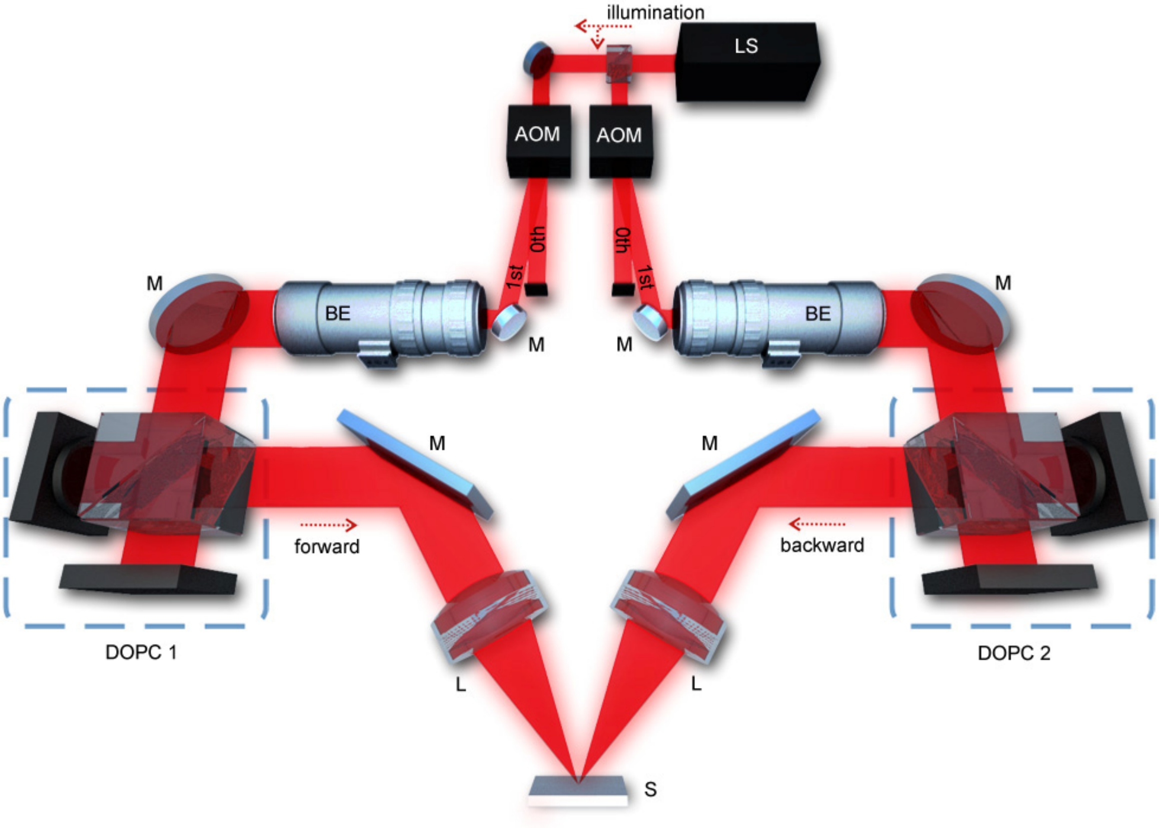

Fig. 3

Experimental setup of the diffusive reflection measurement. LS, laser source; M, mirror; BE, beam expander; L achromatic lens; S, sample. To prepare the sample, we sprayed white color TiO_2 paint onto a glass slide. The paint provided a diffusive surface for the reflection measurement.

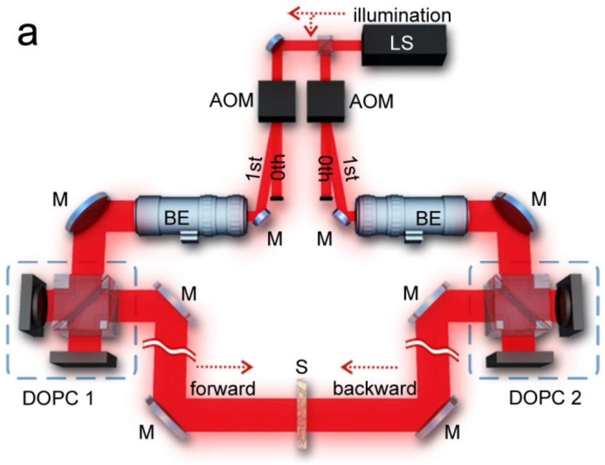

Fig. 4

Experimental setup of the absorber avoidance measurement for the transmission (**a**) and the reflection (**b**) configuration. In both cases, the sample was placed ~0.7 m away from the DOPC system and no focusing lenses were used.

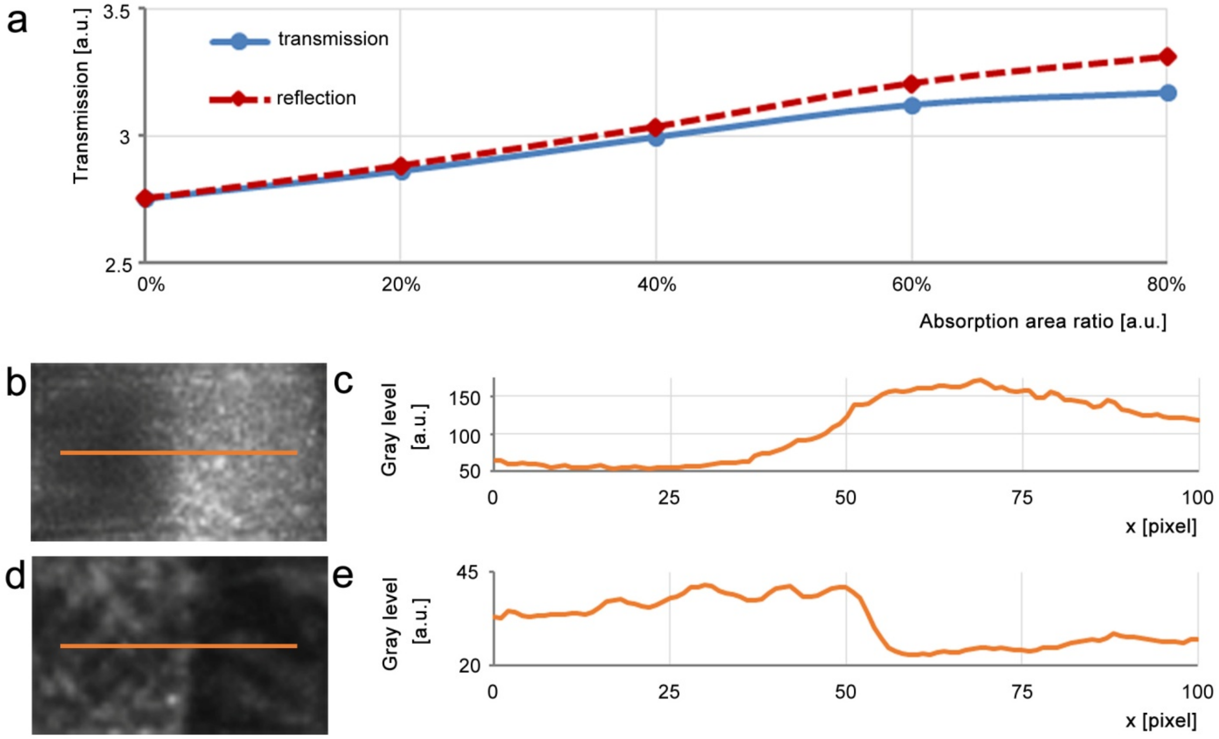

Fig. 5

In the absorber avoidance experiment, the enhancement depended on the absorbance and the area ratio the absorber occupied. In our measurement, the absorbance is ~70% and 75% in the transmission and the reflection configuration, respectively. **a** shows the calculated enhancement after five iterations as a function of the absorption area ratio. The starting point (0%) means that there was no absorber. The enhancement increases at higher absorption area ratio. **b-e** show the cross-sectional view of the light pattern at the end of the iterations in Fig. 3**a** and 3**d** of the main text. The light indeed avoided the absorbers and took the path of higher transmission or reflection.

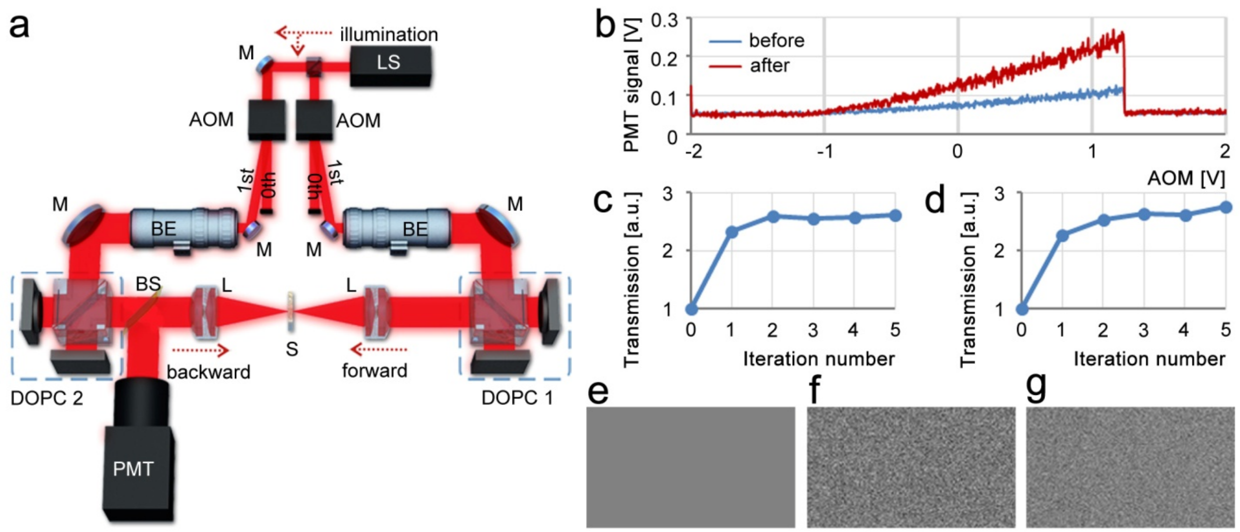

Fig. 6

a Setup of the image transmission experiment. We placed a polarizer and a mask of identical size as the SLM in front of the PMT. The mask and the SLM were at the conjugate plane with respect to the added beam splitter (BS). The polarizer was to ensure that both the PMT and the DOPC measured the same set of optical modes. We also added a lens after the mask to focus the light onto the active area of the PMT. **b** The PMT signal level as a function of the AOM voltage before and after the iterations. **c** The transmission enhancement measured in the analog image transmission experiment. **d** The transmission enhancement measured in the digital image transmission experiment. **e** A uniform image of gray level 128 (half of the maximal level), which was used to measure the SNR of the analog image transmission. **f** Transmitted analog image before the iterations (lower SNR). **g** Transmitted analog image after the iterations (higher SNR).

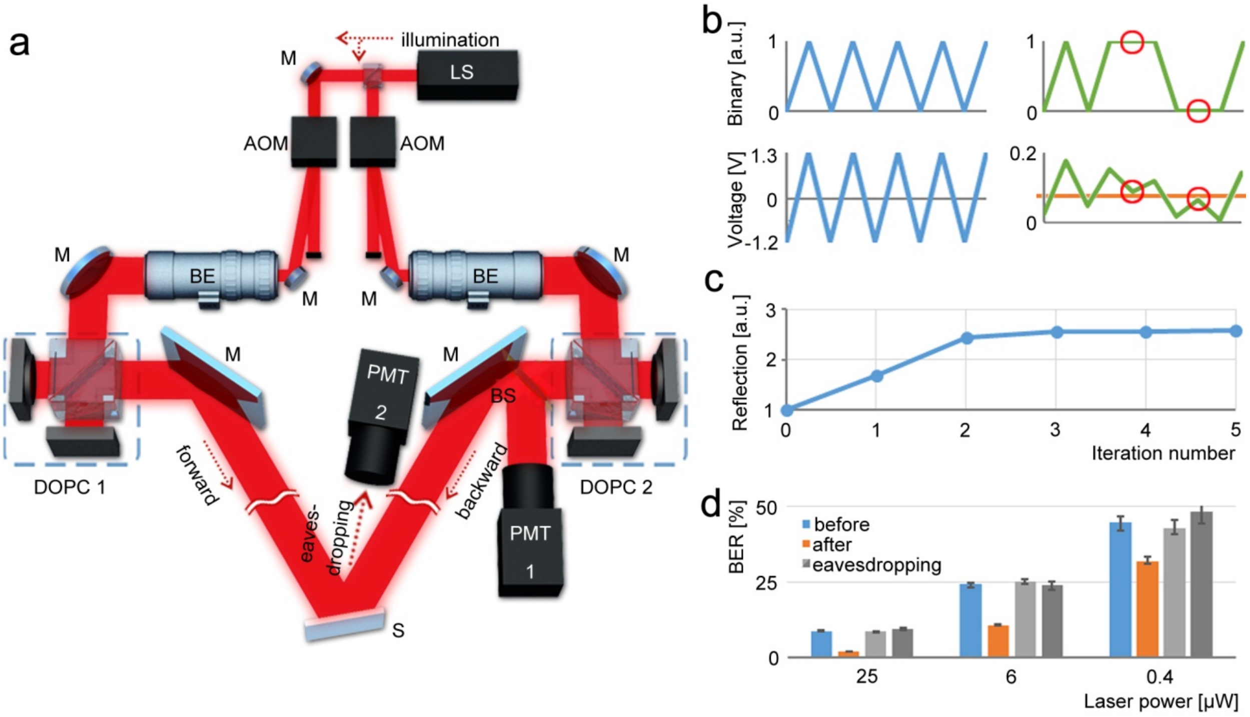

Fig. 7

a Setup of the eavesdropping experiment. We added a second PMT (eavesdropping PMT2) that observed the same diffusive reflection area as the original PMT (PMT1). The diffusive surface is ~0.7 m away from the DOPC. **b** The procedure of the bit-error-rate (BER) measurement. The original data was a "010101…" array. The received binary data was compared to the input data to measure the BER. In the experiment, we switched the AOM voltage between two values (on and off) to modulate the input laser power and converted the received PMT signal to binary data based on a threshold (middle level, orange line). **c** The measured enhancement curve. **d** The measured BER at different input power level. After the iterations, the receiving party's BER (orange bars) was much reduced. However, no improvement was observed for the eavesdropping party (light and dark gray bars). Therefore, the signal enhancement was highly directional and unavailable to the eavesdropping party.

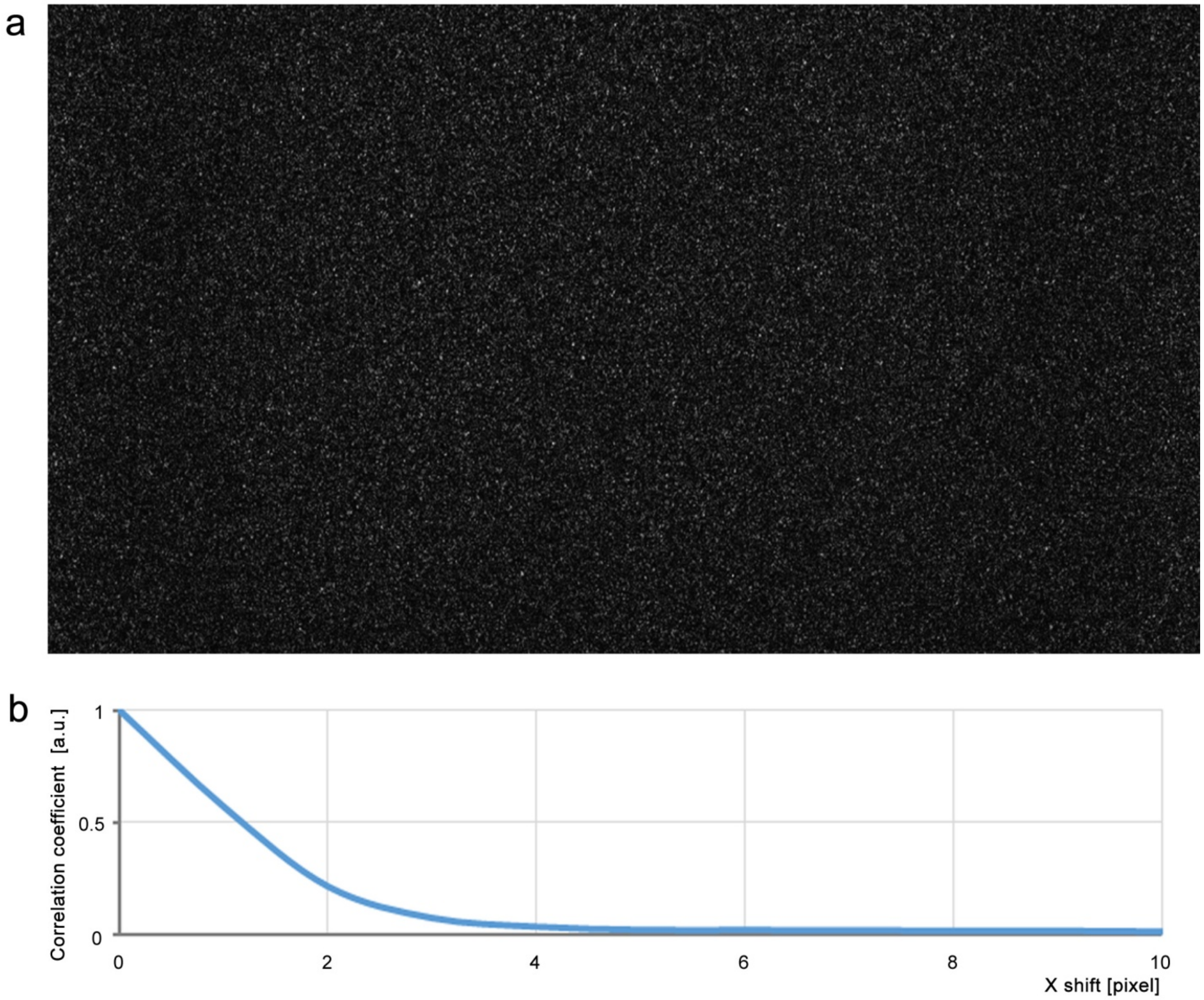

Fig. 8

a A typical speckle pattern observed in the experiments. **b** The autocorrelation of the measured speckle. The full width at half maximum of the autocorrelation was ~ 2 pixels. There were 1920x1080 pixels on the SLM and the number of optical modes was therefore ~5×10^5.
